# MOG-Specific T Cells Lead to Spontaneous EAE with Multilocular B Cell Infiltration in the GF-IL23 Model

**DOI:** 10.1007/s12017-022-08705-2

**Published:** 2022-03-03

**Authors:** Louisa Nitsch, Simon Petzinna, Julian Zimmermann, Daniel R. Getts, Albert Becker, Marcus Müller

**Affiliations:** 1 Department of Neurology, University Hospital Bonn, Campus Venusberg 1, 53127 Bonn, Germany; 2 Department of Microbiology-Immunology and Interdepartmental Immunobiology Center, Northwestern University Feinberg School of Medicine, Chicago, USA; 3 Department of Neuropathology, University Hospital Bonn, Campus Venusberg 1, 53127 Bonn, Germany; 4 School of Molecular Bioscience, University of Sydney, Sydney, Australia

**Keywords:** 2D2 mice, IL-23, Autoimmunity, Neuroinflammation, B cells

## Abstract

Although IL-23 and downstream signal transduction play essential roles in neuroinflammation, the local impact of IL-23 in multiple sclerosis is still not fully understood. Our previous study revealed that the central nervous system (CNS)-restricted expression of IL-23 in a mouse model with astrocyte-specific expression of IL-23, called GF-IL23 mice, leads to spontaneous formation of infiltrates in the brain, especially in the cerebellum. To further investigate the impact of CNS-specific IL-23-expression on neuroinflammation, we studied the GF-IL23 model in mice expressing a myelin oligodendrocyte glycoprotein (MOG)-specific T cell receptor (GF23-2D2 mice). The GF23-2D2 mice developed a chronic progressive experimental autoimmune encephalomyelitis with myelitis and ataxia without requiring additional immunization. CNS-production of IL-23 alone induced pronounced neuroinflammation in the transgenic MOG-specific T cell receptor model. The GF23-2D2 mice spontaneously developed multilocular infiltrates with a high number of B cells, demyelination and a proinflammatory cytokine milieu indicating that the interaction of encephalitogenic T cells and B cells via co-stimulatory factors seemed to be crucial.

**Supplementary Information:**

The online version contains supplementary material available at 10.1007/s12017-022-08705-2.

## Introduction

Multiple sclerosis (MS) is the most common chronic neurological disease in young adulthood leading to permanent neurological deficits. It is a heterogeneous autoimmune disease characterized by inflammatory and demyelinating lesions in the central nervous system (CNS) (Lassmann et al., 2007). The experimental autoimmune encephalomyelitis (EAE) model has served as an animal model of MS for many years. In classical EAE models, EAE is induced either by active immunization against myelin autoantigen suspended in strong immune adjuvants or by injection of preactivated myelin-specific T lymphocytes. These models mimic some immunological aspects of human disease, but do not approach the complexity of human MS. Thus, several different T cell receptor (TCR) transgenic mice have been generated. Mice with myelin oligodendrocyte glycoprotein (MOG)-specific TCR, which are referred to as 2D2 mice, express a transgenic TCR on most CD4 + cells recognizing the MOG 35–55 peptide.

In both mouse models and patients with MS, cytokines were identified as key players in autoimmune diseases. Numerous studies have identified IL-23 as a critical cytokine in the pathogenesis of MS (Javan et al., 2017; Kebir et al., 2009; Sie et al., 2014; Wen et al., 2012). IL-23 is secreted by antigen-presenting cells (APCs) (Oppmann et al., 2000; Pirhonen et al., 2002). It is a heterodimer with a unique p19 subunit that is specific for IL-23 and a common p40 subunit, which is shared with IL-12 (Oppmann et al., 2000). IL-23 receptor signaling contributes to the activation of Th17 cells, which induces the secretion of many cytokines. This subtype of T cells is crucial in chronic inflammation, autoimmune diseases and has been detected in MS lesions (Sie et al., 2014).

Although IL-23 and downstream signal transduction play essential roles in neuroinflammation, the local impact of IL-23 in multiple sclerosis is not fully understood. To decipher the influence of IL-23 on neuroinflammation, we generated a transgenic mouse model with astrocyte-specific expression of IL-23 (GF-IL23) (Nitsch et al., 2019). Our previous study revealed that the CNS-restricted expression of IL-23 enables the spontaneous formation of infiltrates in the brain, especially in the cerebellum. After several months, GF-IL23 mice developed an ataxic phenotype and infiltrates with a high number of lymphocytes, especially B cells. To further elucidate the local impact of IL-23 on neuroinflammation, we backcrossed the GF-IL23 mice with 2D2 mice, called GF23-2D2 mice. In this study, we were able to demonstrate that in GF23-2D2 transgenic mice, IL-23 led to accelerated and pronounced neuroinflammation with ataxia and ascending paraparesis with a chronic course, spontaneous multilocular inflammation with a high number of B cells, demyelination and proinflammatory cytokine response.

## Materials and Methods

### Animals

The generation of transgenic mice with astrocyte-specific expression of IL-23, GF-IL23 mice, has been described in detail previously (Nitsch et al., 2019). MOG 35–55 specific TCR transgenic/ 2D2 mice were obtained from Jackson Laboratories (Bar Harbor, ME, USA) and backcrossed to the GF-IL23 line to obtain hemizygous GF23-2D2 mice. Genotyping of the transgenic offsprings was performed by polymerase chain reaction of tail DNA with a primer that was previously published (Nitsch et al., 2019). All mice were kept under standardized pathogen-free conditions at the animal facility of the University Hospital of Bonn, Germany. Breeding of the animals was approved by the Animal Care Commission of Nordrhein-Westfalen, Germany.

### Clinical Assessment

30–100 days old WT (n = 15), 2D2 (n = 7), GF-IL23 (n = 20), and GF23-2D2 (n = 15) mice were weighed and examined on a daily basis using two different scoring systems, the typical EAE score and an atypical score, as was described previously (Nitsch et al., 2021). The typical EAE score was assessed as follows: 0 no clinical symptoms, 1 limp tail, 2 hind limb weakness, 3 hind limb paralysis, 4 hind and forelimb paralysis, 5 moribund. The atypical EAE score was assessed as follows: 0 no clinical symptoms, 1 hunched appearance, stiff tail, 2 staggered walking, scruffy coat, 3 head tilt, ataxia, obvious impaired balance/ambulation, body lean, 4 inability to maintain upright posture, severe axial rotation, severe body lean, 5 moribund.

### Routine Histology and Immunohistochemistry

Routine histology and immunohistochemistry of symptomatic GF23-2D2 (age 2.5 months) and age-matched 2D2/GF-IL23/WT controls (at least n = 6 per genotype) were performed as described previously (Nitsch et al., 2019, 2021). Infiltration of the cerebellum was measured on cross-sections of H&E stains with a semiquantitative score reaching from 0 to 3 points (0 = no infiltration, 3 = highly infiltrated tissue). Myelinated and demyelinated areas of cerebellum were measured on cross-sections with ImageJ image analysis software to determine the proportion of the demyelinated cebellar area.

### Flow Cytometry

Flow cytometry of symptomatic GF23-2D2 with the age of 2.5 months and age-matched 2D2/GF-IL23/WT controls (6–8 per genotype) was performed as described previously (Nitsch et al., 2019). Fluorochrome-conjugated antibodies (eBioscience; BD Bioscience, Heidelberg, Germany) were used for the detection of CD3e, CD19, CD45. Acquisition was performed using a BD FACSCanto II flow cytometer (BD Biosciences, Heidelberg, Germany). Data were analyzed using the flow cytometry software FlowJo (TreeStar, San Carlos, CA, USA).

### 3′-mRNA Analysis

Transcriptome analysis of symptomatic GF23-2D2 with the age of 2.5 months and age-matched 2D2/GF-IL23/WT controls was performed as described in detail previously (Nitsch et al., 2021). The mRNA of cerebellum homogenates (WT/transgenic mice) was isolated using TRIzol reagent (Invitrogen) according to the manufacturer’s instructions. Library generation for 3′-mRNA sequencing was conducted according to the manufacturer’s guidelines (Quant Seq 3’ mRNA-Seq Library Prep Kit FWD for Illumina; Lexogen). Briefly, 100 ng of total RNA was reverse transcribed by oligo-dT-priming (first strand) and random priming (second strand). After a magnetic bead-based purification step, the libraries were amplified using 15 polymerase chain reaction cycles. Libraries were sequenced on a HiSeq 2500 v4 with a read length of 1 × 50 bp. On average, 10 million raw reads were generated per sample. After trimming the Illumina Universal Adapter with cutadapt reads have been aligned to the mouse genome (STAR). FeatureCounts were used to assign reads to genes based on the definitions of ENSEMBL release GRCm38.94. Inclusion criteria for counting were as follows: uniquely mapped, matching strand, and overlap with only one gene, i.e., non-ambiguous assignment to a single gene. Statistical analysis was performed in the R environment (version 3.5.2) with the Bioconductor R-package DESeq2, with *P* < 0.05 considered significant. Only genes with a total sum of at least two counts across all samples were considered for analysis. Data visualization, such as heatmaps, was generated upon variance stabilizing transformation transferred data using Complexheatmaps.

### Statistical Analysis

Statistical analysis of the clinical assessment and histology was performed using a one-way analysis of variance (ANOVA) or Kruskal–Wallis test followed by an appropriate post hoc test or a Chi-Square test with statistical significance defined as *P < 0.05, **P < 0.01, and ***P < 0.001. Statistical analysis of the flow cytometry was performed using a two-way ANOVA followed by an appropriate post hoc test with *P < 0.05, **P < 0.01, ***P < 0.001. All statistical analyses were performed using GraphPad Prism 5.0 (GraphPad Software).

## Results

### GF23-2D2 Mice Spontaneously Developed an EAE with Myelitis and Ataxia

**Fig. 1:**
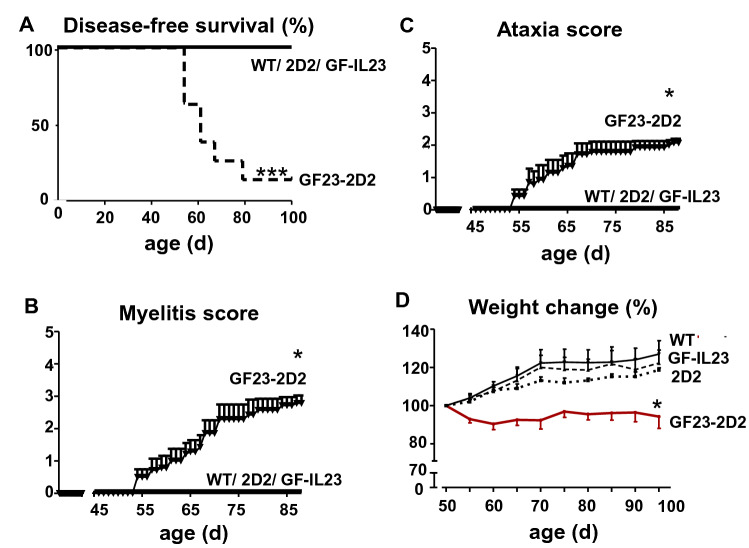
GF23-2D2 mice spontaneously developed myelitis and ataxia. **A**–**C** GF23-2D2 mice spontaneously developed a myelitis and ataxia, whereas WT, 2D2 and GF-IL23 mice did not show any neurological deficits. **D** Consistent with the clinical phenotype, the GF23-2D2 mice showed significant weight loss compared with WT, 2D2, and GF-IL23 mice with *P < 0.05 and ***P < 0.001

In contrast to the WT, GF-IL23, and 2D2 mice, almost all GF23-2D2 (n = 14/15) mice developed neurological deficits in the first 100 days (Fig. 1a, Table 1). GF23-2D2 mice showed a progredient ascending paresis (n = 14/15) (Fig. 1b, Table 1) on average from 61.6 (± 9.3) days onward and a progredient ataxia (n = 13/15) from 62.1 (± 9.4) days onward (Fig. 1c, Table 1). Congruent with the clinical deficits, the GF23-2D2 mice showed a significant weight loss over time (Fig. 1d).

**Table 1:** Clinical features

	Incidence (myelitis)	Incidence (ataxia)	Onset (d) (myelitis)	Onset (d) (ataxia)
2D2	0/7	0/7	–	–
GF-IL23	0/20	0/20	–	–
GF23-2D2	14/15	13/15	61.6 ± 9.3	62.1 ± 9.4

### Multilocular Infiltration in GF23-2D2 Mice

**Fig. 2:**
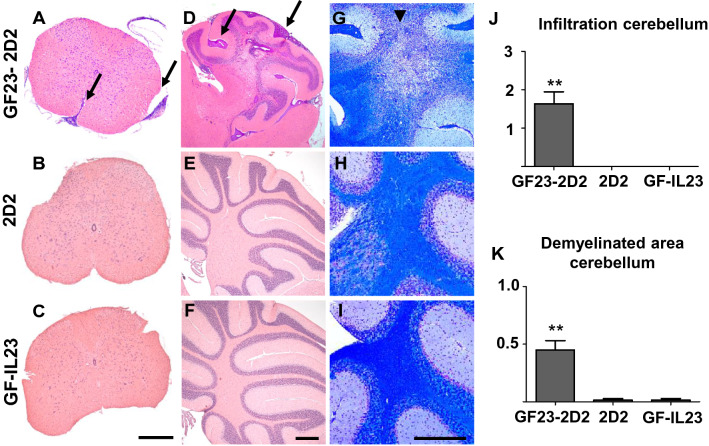
Infiltration and demyelination in GF23-2D2 mice. H&E- (**A**–**F**) and LFB-staining (**G**–**I**) of GF23-2D2, 2D2 and GF-IL23 mice. The myelon (**A**–**C**) show infiltration in GF23-2D2 mice, whereas no infiltrates were detected in 2D2 and GF-IL23 mice. The cerebellum (**D**–**F**) was highly infiltrated in GF23-2D2 mice with both subarachnoidal and intraparenchymal infiltrates as well as diffuse intraparenchymal infiltration. The cerebellum of 2D2 mice (**E**) and GF-IL23 mice (**F**) showed no infiltrates. The LFB-staining displayed demyelinated areas in the parenchyma of the GF23-2D2 cerebellum (**G**) and no demyelination in 2D2 and GF-IL23 mice (H, I). Arrows point to infiltrates, arrow heads to demyelinated areas. The data are representative of at least six transgenic/WT mice. Scale bar: 100 μm. J Infiltration of the cerebellum was measured on cross-sections of H&E stains with a semiquantitative score reaching from 0 to 3 points (0 = no infiltration, 3 = highly infiltrated tissue). GF23-2D2 mice show significantly more infiltrates with **P < 0.01. K Demyelinated area relative to white matter area was quantified in cross sections of cerebellum from GF23-2D2, 2D2 and GF-IL23 mice (n = 4–5 for each group) with **P < 0.01 and showed a significant enhanced demyelination in GF23-2D2 mice

**Fig. 3:**
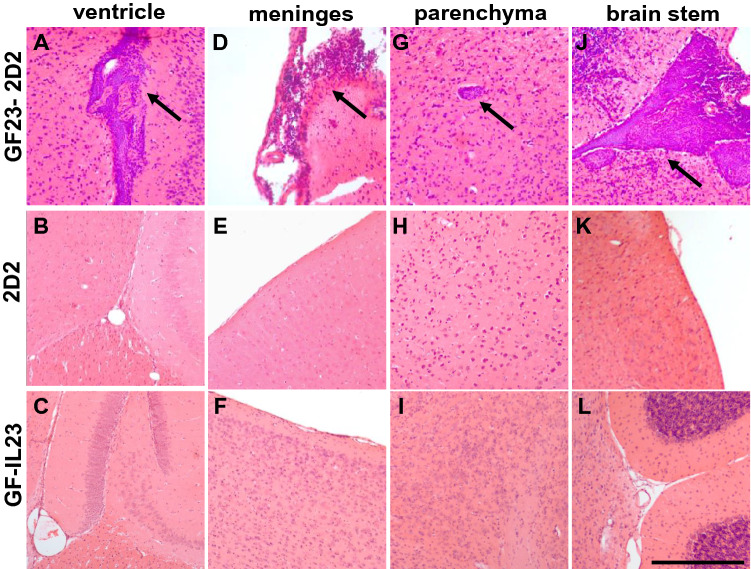
Multilocular infiltration of the CNS in GF23-2D2 mice. Infiltrates in other locations such as the ventricle (**A**–**C**), meninges (**D**–**F**), and parenchyma (**G**–**I**) of the cerebrum and brain stem (**J**–**L**) were found in GF23-2D2 mice but not in 2D2 and GF-IL23 mice. The data are representative of at least six mice of each genotype. Scale bar: 100 μm

Spinal cord infiltrates (Fig. 2a–c) became apparent in the GF-IL23 mice but not in the 2D2 or GF-IL23 mice. The cerebellum of the GF23-2D2 mice was also highly infiltrated (Fig. 2d–f, j). GF23-2D2 mice developed perivascular infiltrates in the subarachnoid space and parenchyma. Additionally, diffuse intraparenchymal infiltration and tissue destruction were observed. Extensive demyelination was noted in GF23-2D2 mice (Fig. 2g–i, k). Furthermore, GF23-2D2 mice developed infiltrates in various other localizations, such as the ventricles, meninges, and parenchyma of the cerebrum and brain stem (Fig. 3). In the 2D2 and GF-IL23 mice, no spontaneous infiltrates were detected in these locations.

### Infiltrates Consisted of Lymphocytes with a Large Proportion of B Cells and were Accompanied by Microglia Activation

**Fig. 4:**
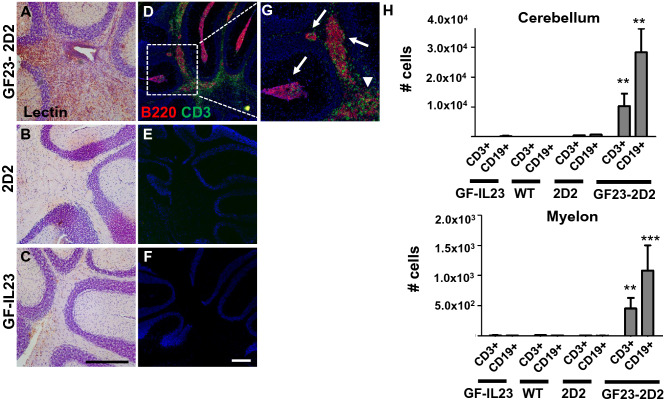
Microglia activation and B cell cluster in GF23-2D2 mice. **A**–**C** Tomato-lectin-staining exhibited activation of microglia in GF23-2D2 mice. Representative data of at least n = 4 mice of each genotype. Scale bar: 100 μm. **D**–**F** higher magnification G Fluorescent immunohistochemistry of GF23-2D2 cerebellum revealed that the infiltrates consisted of lymphocytes with a large number of B cells. There were aggregates with predominant B cell clusters (arrows) surrounded by T cells and a diffuse infiltration of T cells into the parenchyma (arrow heads). Representative data of at least n = 4–6 mice of each genotype. Scale bar: 100 μm. H Flow cytometry data showed consistent findings with predominant CD19 + cells in the spinal cord and cerebellum of GF23-2D2 mice besides CD3 + cells with *P < 0.05, **P < 0.01, and ***P < 0.001

Accompanying the infiltrates, the cerebellum of the GF23-2D2 mice showed strong microglia activation in the lectin stain, whereas this was not evident in the 2D2 and GF-IL23 mice (Fig. 4a-c). Fluorescence double staining illustrated that the infiltrates consisted of T and B cells (Fig. 4d-g). In particular, there were aggregates with predominant B cell clusters surrounded by T cells and a diffuse infiltration of T cells into the parenchyma. The flow cytometry data also emphasized that the infiltrates of the cerebellum as well as of the spinal cord consisted mainly of lymphocytes with a large proportion of B cells (Fig. 4h).

### IL-23 Induced a Proinflammatory Milieu in the 2D2 Model

**Fig. 5:**
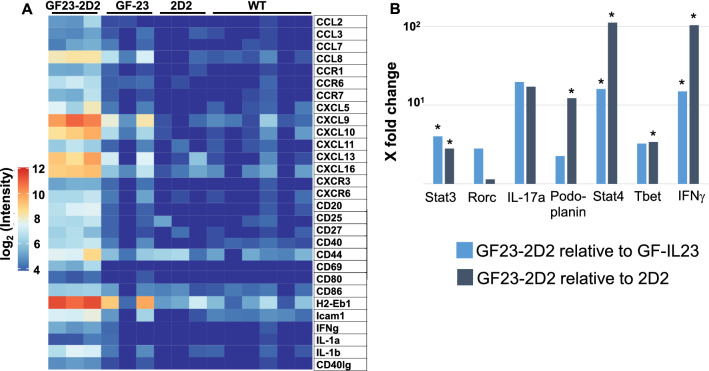
Upregulation of proinflammatory markers and co-stimulatory factors in GF23-2D2 mice A The transcriptome analysis showed the mRNA levels of several proinflammatory markers and co-stimulatory factors of the cerebellum of GF23-2D2, GF-IL23, 2D2, and WT mice. B The mRNA levels of targets involved in Th17 and Th1 cell signaling are listed from GF23-2D2 mice in relation to 2D2 and GF-IL23 with *p < 0.05. *CD* cluster of differentiation, *CCL* chemokine (C–C motif) ligand, *CCR* C–C chemokine receptor type, *CXCL* chemokine (C-X-C motif) ligand, *H2-Eb1* histocompatibility 2, class II antigen E beta, *Icam1* intercellular adhesion molecule 1, *IFNγ* interferon gamma, *IL* Interleukin

For the analysis of activation markers and inflammatory mediators a transcriptome analysis of the cerebellum of the GF23-2D2, GF-23, 2D2, and WT mice was performed (Fig. 5, for statistics Fig. S1). Concerning the chemokines and chemokine receptors, the mRNA levels of CCL2, CCL8, CCR1, CCR6, CCR7, CXCL5, CXCL9, CXCL10, CXCL11, CXCL16, CXCR3, and CXCR6 were increased in the GF23-2D2 mice. The expression markers for T and B cell activation and co-stimulatory molecules, such as CD20, CD25, CD27, CD40, CD40l, CD44, CD86, and ICAM-1, were raised. IFNγ, IL-1b were upregulated. The expression of CXCL13 and H2-Eb1/MHCII was increased in the GF23-2D2 mice, but the increase was significant only when compared to the WT and 2D2 mice, not to the GF-IL23 mice. Figure 5b lists the expression of the typical downstream targets involved in Th17 and Th1 cell signaling in GF23-2D2 compared to GF-IL23 and 2D2 mice. Regarding Th17 cell signaling, signal transducer and activator of transcription (Stat) 3 and Podoplanin were significantly elevated in GF23-2D2 mice. IL-17a or RAR-related orphan receptor C (Rorc), which encodes RAR-related orphan receptor gamma (Rorγ), were increased, but not significantly. In contrast, T box expressed in T cells (T bet), Stat 4 and IFNγ, all involved in Th1 cell signaling, were significantly upregulated in GF23-2D2 mice.

## Discussion

IL-23 is an essential cytokine in the development of MS. In our previous work, we demonstrated that astrocyte-specific IL-23 secretion in the GF-IL23 model leads to spontaneous formation of infiltrates after approximately 6 months, especially in the cerebellum with a high proportion of B cells (Nitsch et al., 2019). To further investigate the impact of CNS-specific IL-23-expression on neuroinflammation, we studied the GF-IL23 model using a 2D2 background. We examined whether the B cell-mediated mechanisms of neuroinflammation in the GF-IL23 model were, without prior immunization, sufficient for inducing EAE on the 2D2 background.

A proportion of 2D2 mice spontaneously develop isolated optic neuritis without clinical or histological manifestation of EAE, which was in contrast to the GF23-2D2 model (Bettelli et al., 2003). Thus, the GF23-2D2 model stands out in several respects. Here, the astrocyte-specific IL-23 expression together with autoreactive T cells resulted in the development of a severe and spontaneous EAE in almost all mice without prior immunization. Additionally, there was no remission of the deficits, but rather a chronic course. The GF23-2D2 mice not only developed infiltrates in the myelon, but also showed a multilocular manifestation with infiltration of the cerebellum, the parenchyma of the forebrain, the ventricles and the brain stem. Furthermore, the GF23-2D2 mice showed a pronounced demyelination in the cerebellum.

In the GF23-2D2 mice, multilocular and severe infiltration with a high proportion of B cells and demyelination resembled the EAE model in the GF-IL23 mice after immunization with the MOG35-55 peptide, as we have previously described; however, no adjuvans and immunization were needed to drive this strong neuroinflammatory response in the GF23-2D2 mice (Nitsch et al., 2021). CNS-produced IL-23 alone was sufficient to drive pronounced neuroinflammation, demyelination and tissue destruction in our model. In WT EAE mice, IL-23 produced by CNS-resident cells controls T cell encephalitogenicity during the effector phase (Becher et al., 2003). However, previous clinical trials of anti-IL23 antibody treatment in MS have not been promising. Administration of the p40 antibody ustekinumab was unsuccessful in patients with MS (Segal et al., 2008). Similarly, the anti-p40 antibody briakinumab showed only a slight benefit in terms of imaging progress and clinical relapse rate (Vollmer et al., 2011). IL-23 could be more important at the beginning of disease development and not at later disease stages, when the disease has already manifested. Further, the anti-p40 antibodies used could have had limited access to the CNS and could not act at the site where IL-23-driven neuroinflammation occurs. The GF23-2D2 model could be a model for investigating this further.

B cell accumulation and the B cell follicle like infiltrates are striking in the GF23-2D2 model. B cells and plasma cells are essential components of MS lesions. Their accumulation and leptomeningeal inflammation with ectopic lymphoid follicles were detected in patients with MS (Lucchinetti et al., 2011; Serafini et al., 2004). Characteristic findings in the cerebrospinal fluid (CSF) in MS are oligoclonal immunoglobulin bands indicating antibody production in the CNS. Thus, immunotherapies targeting B cells are established for the treatment of MS. Peters et al. describe that Th17 cells induce ectopic lymphoid follicles in the CNS and that this is partly dependent on IL-17 and Podoplanin (Peters et al., 2011). IL-17a and Podoplanin, both involved in Th17 cell signaling, were upregulated in the cerebellum of GF23-2D2 mice, but only the latter significantly.

Other MOG-specific T cell transgenic models have been used to investigate the significance of B cells in neuroinflammation. Bettelli et al. (2006) crossed the TCR MOG mice with MOG-specific Ig heavy-chain knock-in mice, in which 60% of the mice spontaneously developed a severe form of an EAE. The model emphasized the interaction of B and T cells. They found class switching of the immunoglobulins to IgG1 in the presence of the transgenic T cells. MOG-specific B cells enhanced MOG-specific T cell proliferation and activation in this model. In contrast, in the GF23-2D2 model, astrocyte-specific secretion of IL-23 with autoreactive T cells alone were sufficient to drive neuroinflammation. One could speculate that B cells in the GF23-2D2 model were already activated and autoreactive due to IL-23 to drive neuroinflammation in 2D2 mice.

Another transgenic EAE model with expression of a T cell receptor specific for the MOG peptide 92–106 in the context of MHC class II on a SJL/J background led to the spontaneous development of EAE and further clarified the interaction between B and T cells in this mainly T cell-driven model (Pöllinger et al., 2009). These mice expressed a transgenic TCR without transgenic MOG-specific B cells although autoreactive B cells expanded during the disease and produced pathogenic antibodies against MOG epitopes, thereby enhancing the EAE (Pöllinger et al., 2009). Therefore, autoreactive T cells seemed to interact with endogenous B cells, which led to the expansion of MOG-specific autoreactive B cells from the endogenous repertoire.

A similar pathomechanism may be crucial in the GF23-2D2 model. The high expression of class II MHC and co-stimulatory molecules like CD27, CD40, CD40L, CD86 implicate that the B cells in our model drive neuroinflammation via co-stimulatory factors. CD27 is a regulator of B cell activation and immunoglobulin synthesis, and CD40/CD40L acts as an inductor of B cell proliferation, generation of memory B cells, blocks cell apoptosis, and mediates antibody class switching (Andre et al. 2002). The importance of the interaction of B and T cells during neuroinflammation was demonstrated in many studies. Antigen-specific B cells induce naïve CD4 + cell proliferation *in* *vivo* (Townsend et al. 1998), and the role of B cells as APCs in autoimmune disease was proven (Waisman et al. 2018). Furthermore, the interaction between antigen-specific T and B cells is necessary for B cell maturation and immunoglobulin isotype class switching. In addition, T cells are essential for the differentiation of B cells into Ig-secreting plasma cells or long-lived memory B cells.

Besides the upregulation of co-stimulatory factors and activation markers, the transcriptome analysis of the GF23-2D2 mice revealed a proinflammatory milieu with broad upregulation of cytokines in the cerebellum. The high expression of IFNγ, IL-1b, chemokines, and chemokine receptors in the transcriptome analysis were striking and in line with our previous study of MOG35-55-induced EAE in GF-IL23 mice (Nitsch et al., 2021). Among them, CCL8, CXCL9, CXCL10 CXCL11, CXCL13, and CXCL16 are involved in the chemotaxis of immune cells (Dhaiban et al., 2020, Trebst et al. 2001). CXCL13 is involved in B cell trafficking (Ferretti et al., 2016) and is increased in the meninges and CSF of patients with MS. Elevated CSF levels correlate with the B cell count in the CSF, markers of immune activation, disease activity, and gray matter damage (Magliozzi et al., 2018; Sellebjerg et al., 2009).

Regarding Th17 cell signaling, only Stat3 was significantly upregulated in our study; Rorc and IL17a were elevated but not significantly. This indicates that the IL-23 mediated mechanism of immune cell accumulation and aggravation of the local proinflammatory cytokine milieu may be partly independent of the Th17 cell axis. In our study we found elevation of the Th1 cell associated Stat4, Tbet besides IFNγ in GF23-2D2 mice. The role of Th1 cells in the model with astrocyte-specific expression of IL-23 is in line with our previous study of the spontaneous course of the GF-IL23 mice and the study about MOG35-55 EAE in GF-IL23. There we found also increased levels of IFNγ and also Th1 cells in the cerebellum, but also IL-17a and IFNγ co-expressing CD4 + cells in older mice (Nitsch et al., 2019). In MS patients, the levels of IFNγ are associated with the disease activity (Kaskow, Baecher-Allan 2018) and the myelin-reactive T cell repertoire produces IFNγ in response to antigens (Olsson, 1992). By increasing the expression of MHC molecules, IFNγ as well enhances the antigen-presentation of myeloid cells, which in turn results in activation of myelin-antigen-specific CD4 + or CD8 + cells (Kaskow, Baecher-Allan 2018).

Taken together, the MOG-specific transgenic T cell model in GF-IL23 mice led to a chronic progressive EAE without additional immunization. CNS-produced IL-23 expression alone in the MOG-specific transgenic T cell mouse model was sufficient to induce pronounced neuroinflammation. The GF23-2D2 mice developed spontaneous formation of multilocular infiltrates with a high number of B cells, demyelination and a proinflammatory cytokine milieu. The interaction of encephalitogenic T cells and B cells via co-stimulatory factors seemed to be important in the GF23-2D2 model.

## Supplementary Information

Below is the link to the electronic supplementary material.

Supplementary file1 (PDF 68 KB)

## Data Availability

Data will be made available upon reasonable request.
