# Supplementary material for: MOG-Specific T Cells Lead to Spontaneous EAE with Multilocular B Cell Infiltration in the GF-IL23 Model
Source: Neuromolecular Med. 2022 Mar 3;24(4):415–23. doi: 10.1007/s12017-022-08705-2 (PMC9684240; doi:10.1007/s12017-022-08705-2)
Supplement: Supplementary file 1 — Supplementary file1 (PDF 68 KB) [file 12017_2022_8705_MOESM1_ESM.pdf]

	GF23-2D2/2D2		GF23-2D2/WT		GF23-2D2/GF23	
	xlog2	p value	xlog2	p value	xlog2	p value
CCL2	7.41	1.3E-05	6.85	3.6E-07	5.86	0.00023
CCL3	4.23	0.012	5.17	0.00035	2.66	0.069
CCL7	6.62	0.00028	5.24	0.00023	2.35	0.10
CCL8	8.09	8.1E-11	6.50	3.1E-13	1.90	0.046
CCR1	6.31	8.5E-05	6.72	1.0E-07	4.47	0.00047
CCR6	8.04	1.2E-08	7.01	5.6E-14	4.98	2.0E-08
CCR7	8.03	6.9E-07	6.08	1.9E-07	5.53	3.2E-05
CXCL5	9.78	4.2E-11	5.93	6.9E-12	5.10	1.2E-07
CXCL9	9.84	1.1E-10	6.74	1.6E-08	3.00	0.028
CXCL10	10.51	4.2E-11	6.53	1.5E-11	3.43	0.0015
CXCL11	5.10	6.0E-05	5.62	3.2E-07	4.16	0.00046
CXCL13	5.39	0.016	8.03	4.8E-05	2.61	0.24
CXCL16	5.81	1.3E-09	5.59	8.6E-12	3.41	0.00022
CXCR3	7.03	9.6E-05	5.67	5.6E-05	3.06	0.033
CXCR6	7.79	1.2E-06	5.95	3.7E-08	3.17	0.0046
CD20	9.51	8.0E-12	7.48	1.5E-16	6.18	2.1E-11
CD25	3.63	0.011	6.17	3.3E-06	5.50	0.00023
CD27	5.06	2.1E-05	6.21	9.4E-09	5.46	5.6E-06
CD40	8.86	9.2E-10	5.28	2.4E-10	3.82	1.9E-05
CD44	2.44	0.0064	3.93	5.3E-07	2.98	0.0009
CD69	7.86	0.096	7.78	0.054	8.15	0.084
CD80	4.17	0.23	4.10	0.16	2.58	0.45
CD86	2.93	0.0021	3.82	6.0E-06	2.49	0.0076
H2-Eb1	5.35	7.4E-05	6.16	1.5E-07	2.32	0.085
Icam1	8.79	3.1E-09	4.34	9.7E-08	2.67	0.0035
IFNg	6.69	0.0016	5.35	0.0017	3.95	0.036
IL-1a	4.17	0.17	3.62	0.16	1.19	0.68
IL-1b	7.51	3.2E-08	6.47	6.9E-12	4.45	5.1E-06
CD40lg	6.16	0.0011	5.60	0.00022	4.03	0.017
